# Novel Interactions between Actin and the Proteasome Revealed by Complex Haploinsufficiency

**DOI:** 10.1371/journal.pgen.1002288

**Published:** 2011-09-22

**Authors:** Brian Haarer, Dimitra Aggeli, Susan Viggiano, Daniel J. Burke, David C. Amberg

**Affiliations:** 1 Department of Biochemistry and Molecular Biology, Upstate Medical University, State University of New York, Syracuse, New York, United States of America; 2 Department of Biochemistry and Molecular Genetics, University of Virginia Medical Center, Charlottesville, Virginia, United States of America; Stanford University School of Medicine, United States of America

## Abstract

*Saccharomyces cerevisiae* has been a powerful model for uncovering the landscape of binary gene interactions through whole-genome screening. Complex heterozygous interactions are potentially important to human genetic disease as loss-of-function alleles are common in human genomes. We have been using complex haploinsufficiency (CHI) screening with the actin gene to identify genes related to actin function and as a model to determine the prevalence of CHI interactions in eukaryotic genomes. Previous CHI screening between actin and null alleles for non-essential genes uncovered ∼240 deleterious CHI interactions. In this report, we have extended CHI screening to null alleles for essential genes by mating a query strain to sporulations of heterozygous knock-out strains. Using an *act1Δ* query, knock-outs of 60 essential genes were found to be CHI with actin. Enriched in this collection were functional categories found in the previous screen against non-essential genes, including genes involved in cytoskeleton function and chaperone complexes that fold actin and tubulin. Novel to this screen was the identification of genes for components of the TFIID transcription complex and for the proteasome. We investigated a potential role for the proteasome in regulating the actin cytoskeleton and found that the proteasome physically associates with actin filaments *in vitro* and that some conditional mutations in proteasome genes have gross defects in actin organization. Whole-genome screening with actin as a query has confirmed that CHI interactions are important phenotypic drivers. Furthermore, CHI screening is another genetic tool to uncover novel functional connections. Here we report a previously unappreciated role for the proteasome in affecting actin organization and function.

## Author Summary

Individuals inherit two copies of each gene, one from each parent, and frequently the two copies are different from each other. Sometimes one copy is completely defective, but since there is one normal copy there may be no negative consequences. Our research is focused on understanding the consequence of inheriting one bad copy for two or more different genes. Geneticists refer to inheriting one bad copy of a gene as a haploinsufficiency and inheriting one bad copy of multiple genes as a complex haploinsufficiency. Using yeast as a model system, we have addressed, in a systematic way, what occurs if a cell inherits one bad copy of the gene called actin and one bad copy of one of the ∼1,000 essential genes in the yeast genome. We discovered that complex haploinsufficiency between actin and one of 60 different essential genes leads to reduced cell viability. These 60 genes are highly enriched for certain functional groups, including those involved in protein degradation and gene expression. It is expected that approaches such as these in model organisms will be applicable to understanding the potential for deleterious complex haploinsufficient interactions in the human genome.

## Introduction

There has been an increasing interest in multi-genic influences on human disease, perhaps in part because most genetic disorders with simple, monogenic origins have been described but also because some very prevalent and devastating diseases, such as cancer and psychiatric disorders, are now recognized to have complex genetic influences [1]. Extensive efforts are being made to identify the genes involved in complex genetic disorders, but this frequently amounts to trying to find a needle in a haystack of genetic diversity. An admittedly large chasm exists between these top-down approaches (disease-to-gene) and the bottom-up approaches (gene-to-phenotype) being leveraged in model systems, but an argument can be made that the bottom-up approaches are having a broader impact in how we think about genome dynamics and hold the promise for identifying conserved genetic vulnerabilities. For example, the large datasets generated by yeast genomic and proteomic approaches have been critical to the field of network biology and have helped lead to an understanding of system robustness, interconnectedness and the evolutionarily conserved aspects of biological networks [2]. The relatively high number of connections between genes and proteins in yeast biological networks suggests that systems can often adjust to the loss of a single component, a prediction that is born out by the simple observation that most yeast genes (∼5,000 out of ∼6,000) are not essential [3]. Secondly, yeast networks show short path lengths between all genes/proteins in the network, indicating a high level of interconnectedness between cellular functions [4]. Clearly the greatest contributions to understanding genetic interaction networks has come from the labs of Charles Boone and colleagues in their efforts to define the entire constellation of binary synthetic lethal interactions by synthetic genetic array (SGA) analysis in *Saccharomyces cerevisiae*. Besides contributing to our understanding of biological network topology, the extensive amount of genetic interaction data generated by SGA synthetic lethal screening has allowed for understanding epistatic relationships and how they can be interpreted to infer functional relatedness, and in particular whether sets of genes function in the same, parallel, or unrelated pathways [4]–[5].

Our lab has more recently become interested in digenic influences on phenotype, specifically in diploid cells and organisms. Our approach is distinguished from that of SGA analysis because it allows for complications resulting from heterozygosity at two loci. Specifically, we examine the phenotypic consequences of being hemizygous (one loss of function allele/one wild type allele) at two loci; if the negative consequence is greater than that expected based on single hemizygosity, we define the gene pair as displaying a complex haploinsufficient interaction (CHI). Recent population-scale sequencing of human genomes suggests to us that CHI interactions may be very important phenotypic drivers and contributors to genetic disorders in human populations. The 1000 Genomes Project Consortium recently published the results from whole-genome sequencing of 179 individuals and remarkably discovered that on average, human individuals inherit 250–300 loss-of-function alleles [6]. This is a strikingly high number in comparison to previous estimates and suggests that every person inherits a unique combination of over 44,000 potentially deleterious CHI gene pairs (300-choose-two is a binomial coefficient determined by the factorial equation N!/K!(N-K)! where N is the number of elements and K the subsets of N). A critically important question is how many complex hemizygosities are deleterious. We previously addressed this question using the yeast model and a null allele of the essential actin gene to screen for CHI interactions with the ∼4,800 non-essential gene knock-out alleles. We have found that null alleles for ∼240 non-essential yeast genes display deleterious CHI interactions with the actin null allele ([7]. Although we admit that actin is a particularly important and multi-functional gene, these results strongly argue that the landscape of potentially deleterious CHI interactions is likely to be quite large.

As has been observed in the synthetic lethal data, our network of CHI genes is highly enriched for certain functional groups including several functions and complexes known to be involved in actin function. In addition, we uncovered unanticipated genetic vulnerabilities reflecting novel connections to the actin cytoskeleton that were not predicted by more traditional approaches such as protein interaction studies [7]. What this suggests to us is that efforts to map the causes of multi-genic human disorders is going to continue to be problematic as it may be difficult to predict which genes neighboring a chromosomal region linked to disease inheritance are responsible for the phenotype of interest. This is likely to be made even more complicated by the influences of several hundred additional loss-of-function alleles. We argue that knowing the landscape of binary CHI gene interactions in a simple eukaryotic may be very useful in predicting multi-genic influences in human genetic disorders, in particular when the affected genes disrupt core cellular functions. At this time we do not have clear examples of CHI interactions causing human disease. However, single gene haploinsufficiencies in 32 different transcription factors causes a diverse array of human genetic disorders [8], haploinsufficiency of 23 different tumor suppressor genes has been shown to contribute to tumorigenesis [9], and complex haploinsufficiency has been shown in mouse models to contribute to early aging [10] and tumorigenesis [11]–[12]. We suspect that examples of CHI interactions in humans are lacking due to the difficulties associated with detecting these interactions and not because they aren't consequential.

Since our first CHI screens were performed on the non-essential gene set, many core and conserved cellular processes that are facilitated largely by essential genes were missed. Here we report an adaptation of this approach that enables the analysis of complex heterozygous combinations of essential gene deletions and its use to expand the actin CHI network to include 60 essential genes. There is extremely significant functional enrichment in the actin CHI essential gene network, including the inclusion of many genes for proteasome components. The proteasome is known for its ability to degrade ubiquitinated proteins. However, our preliminary analysis of its role in actin regulation suggests it may play a structural role in the actin cytoskeleton.

## Results

### A novel screen for the identification of complex haploinsufficient interactions between an actin null allele and null alleles of the essential genes of yeast

In a previous report [7], we presented a genome-wide analysis of complex haploinsufficiency (CHI) interactions arising in diploid strains deleted for one copy of the unique and essential actin gene (*ACT1*) and for one copy of a non-essential gene. This was performed by mating a haploid strain deleted for *ACT1* (kept alive by a counter-selectable *ACT1*-containing plasmid) with the EuroScarf haploid non-essential gene deletion mutant array. Diploids that were impaired for growth without the *ACT1* plasmid indicated that the double hemizygosity was incapable of supporting (robust) growth. While we have uncovered ∼240 such detrimental combinations ([7]; and Haarer, Viggiano and Amberg, unpublished), an argument can be made that perhaps more relevant or stronger CHI interactions might occur when *both* hemizygous mutations are in essential genes. Furthermore, many fundamental processes are executed by essential genes and these would have been missed by the previous screening method.

In order to carry out a similar screen against essential genes, we took advantage of the observation that spores carrying deletions of many essential genes are still competent to mate prior to dying [13], presumably due to a sufficient complement of maternal factors to support germination and limited growth. Therefore, we first sporulated the hemizygous diploids from the EuroScarf (http://web.uni-frankfurt.de/fb15/mikro/euroscarf/) collection of *S. cerevisiae* strains deleted for essential genes. These spores were then directly mated to our *act1*Δ query strain (carrying *ACT1* on a counter-selectable plasmid) and diploids carrying both the Nat^R^-marked *act1*Δ allele and G418^R^-marked gene deletions were selected and analyzed for growth defects on medium that selected for cells that had lost the *ACT1*-bearing plasmid. In this manner we were able to perform complex haploinsufficiency screens against these essential genes.

As with CHI screening against non-essential genes [7], our essentials-only CHI screens generated substantial numbers of interactions that varied in severity from lethal to moderately slow growing. Overlap between screens varied, ranging from 11 to 49%. From four separate screens against the EuroScarf collection of 1095 essential-gene deletion strains, we obtained a total of 212 potential CHI interactions, of which 39 were hit twice, 14 were hit three times, and 6 were hit in all four screens.

**Figure 1:**
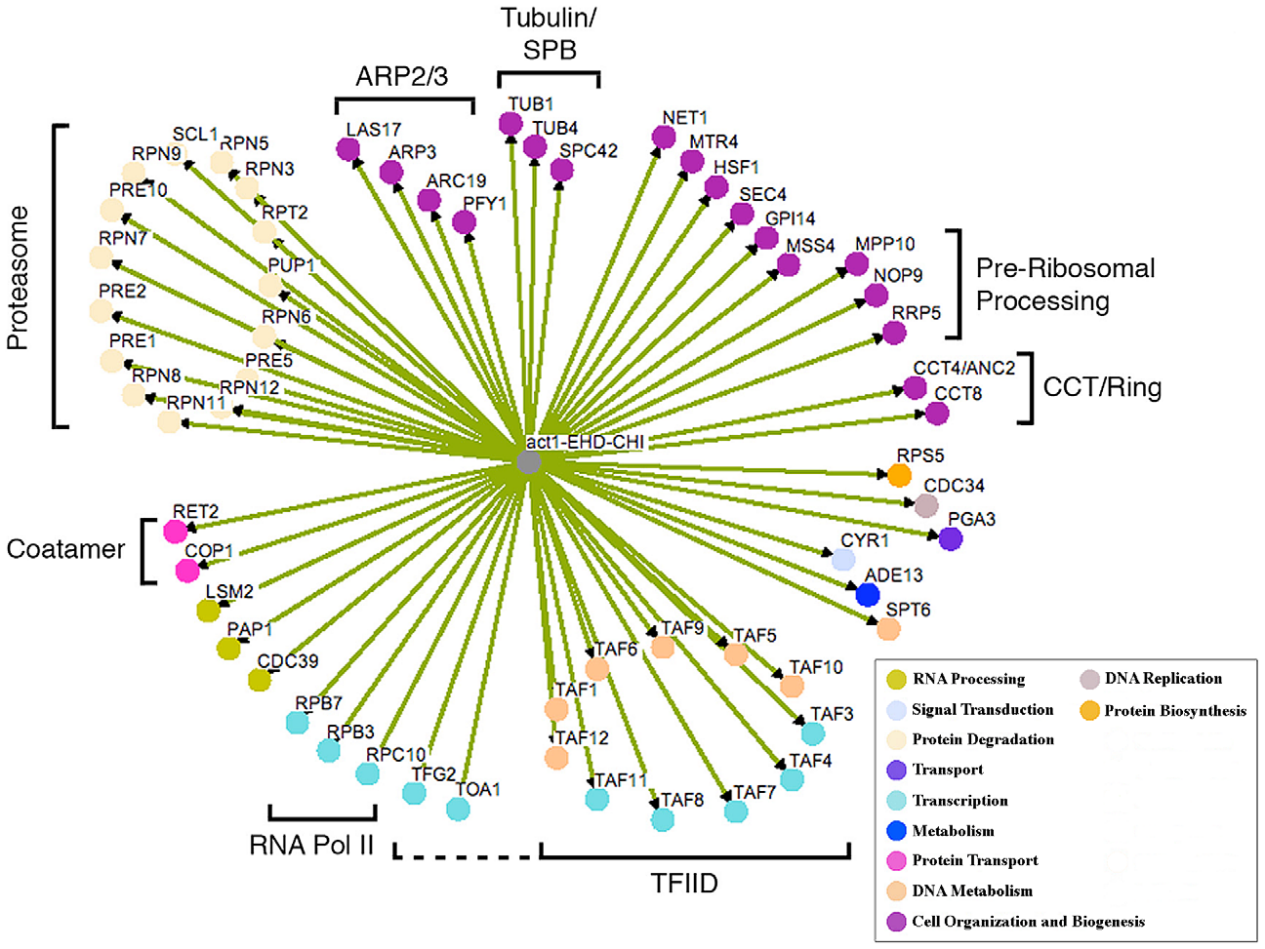
Network of CHI interactions between *act1Δ* and null alleles for essential genes. Where applicable, genes are grouped into functional categories; color-coding of major GO terms is as indicated in the key. The interaction network was generated using the program Osprey [46]. EHD, essential heterozygous diploid.

**Figure 2:**
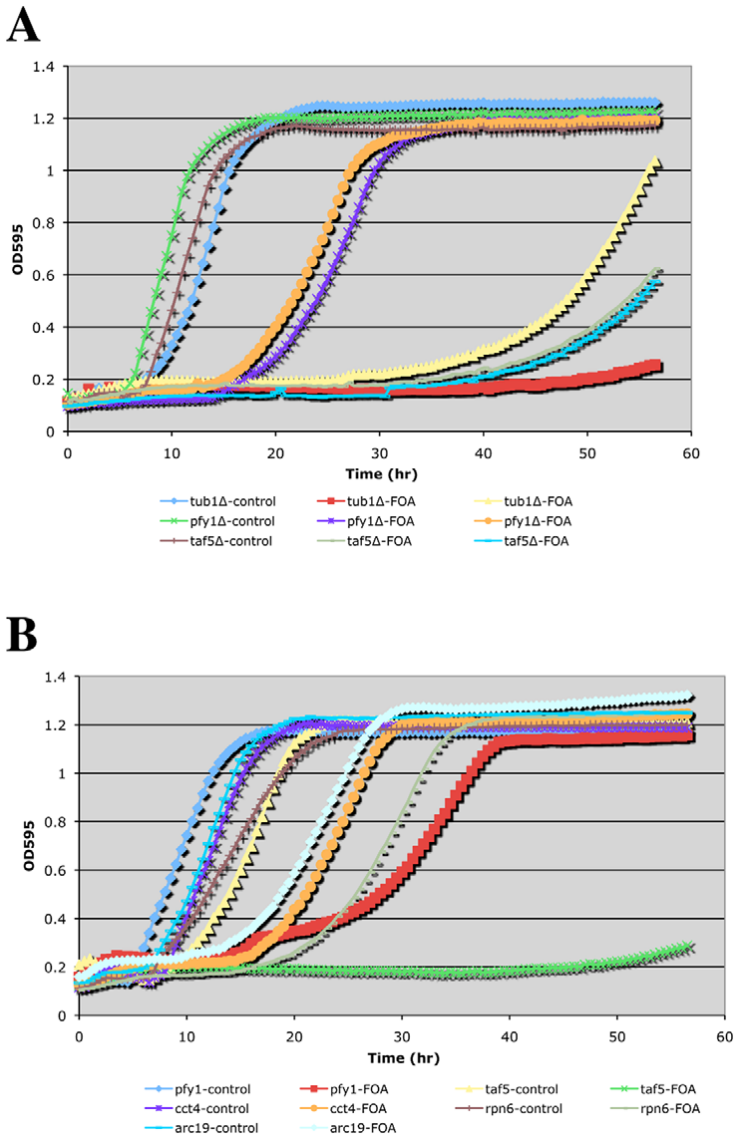
Growth curves of doubly hemizygous strains. A TECAN shaking incubator/microplate reader was used to follow growth of the indicated complex hemizygotes (with *ACT1/act1Δ::Nat^R^*) in SC^msg^+G418+Nat media without (control) or with FOA. Panels (A) and (B) are from independent experiments.

We have previously found that screening robotically results in a large number of false positives necessitating manual confirmation or “hand testing” which involves manually mating the strains on plates and streaking for single colonies on selective plates. This allows us to examine the growth characteristics of several clonal isolates for each CHI cross. Hand retesting of the 212 potential CHI interactions allowed us to confirm 48 authentic *act1Δ* CHI interactions; the other 164 failed to confirm by the more rigorous hand tests and are therefore false positives. From the primary screen data, we identified related functional groups (see below) that implicated another 24 genes; of these, 12 of the genes' null alleles tested positive for a CHI interaction in subsequent hand tests. This combined network of 60 genes is shown in Figure 1; note that the color coding reflects the major functional gene ontology terms assigned to the genes as indicated in the key. This number of interactions is probably underestimated, as we continue to see some variability or “escape” from the FOA screen (see below and Figure 2A). We believe that escape can occur through multiple mechanisms, including conversion or duplication at either mutant locus, mutational inactivation of the plasmid-borne *URA3* marker, or chromosome non-disjunction leading to trisomies for either the chromosome bearing the CHI gene or a chromosome for some other gene capable of suppressing the CHI interaction. We believe the latter is most likely, as the reported frequency of spontaneous aneuploidies in *S. cerevsiae* is as high as 1×10^−4^ [14]–[15]. Notably, the number of confirmed hits for both the essential and non-essential deletion collections is ∼5% of the total number of genes represented in each collection, which would seem to suggest that the essential genes are no more predisposed to CHI interactions than are the non-essential genes. However, it may be that that the false negative rate is higher for the essential gene screens possibly as a result of the sporulation step; if this is true then essential genes may, on average, have more CHI interactions than non-essential genes as has been observed for synthetic lethal screens.

### Phenotypic analysis of CHI strains

To obtain a more quantitative measure of CHI interactions, we sought to determine growth rates of double hemizygotes upon loss of the *ACT1* plasmid. For this purpose we adopted a small-scale liquid media approach and utilized a TECAN microplate reader/shaking incubator. Several notable observations were made while carrying out these liquid media growth tests: 1) The restrictive temperature for actin hemizygotes is significantly lower in liquid media. While 34–35° (our typical “high temperature” CHI screening mark) is permissive for growth of *ACT1/act1Δ* strains on solid media, we found that growth above 30° was severely impaired for such strains in liquid media. 2) Only the strongest CHI hits (generally scored as 1 = inviable or 2 = severe growth defects) exhibited detectably reduced growth rates by this method (see Figure 2B). Presumably, expansion of the CHI culture allows for greater opportunities for suppression events caused by genetic instability such as the generation of trisomies by chromosome non-disjunction. As mentioned above, even for the strongest CHI interactions, such as the interaction with *tub1Δ*, escape events are not infrequent. This can be seen fairly clearly in Figure 2A where the *ACT1/act1Δ tub1Δ/TUB1* complex hemizygote culture traced with yellow triangles had an escape event while the culture traced in red squares did not. Interestingly, the liquid growth behavior of CHI strains manifested as delays in the onset of logarithmic growth and/or differences in the slopes during logarithmic growth (see Figure 2A and 2B). For the strongest CHI interactions, such as with *tub1Δ* and *taf5Δ*, long delays are followed by low slopes in the growth curve. For CHI interactions of more modest strength, such as with *pfy1Δ*, *cct4Δ*, and *arc19Δ*, modest delays are followed by fairly small differences in slope from the control cultures. Curiously, the *rpn6Δ* culture shows a delay over the control culture but no difference in slope once log-phase growth is entered (Figure 2B). Although it is difficult to obtain a strict quantitative measurement of a CHI interaction using TECAN growth curve analysis, it does provide another means to compare the strengths of the interactions over mere colony size.

**Figure 3:**
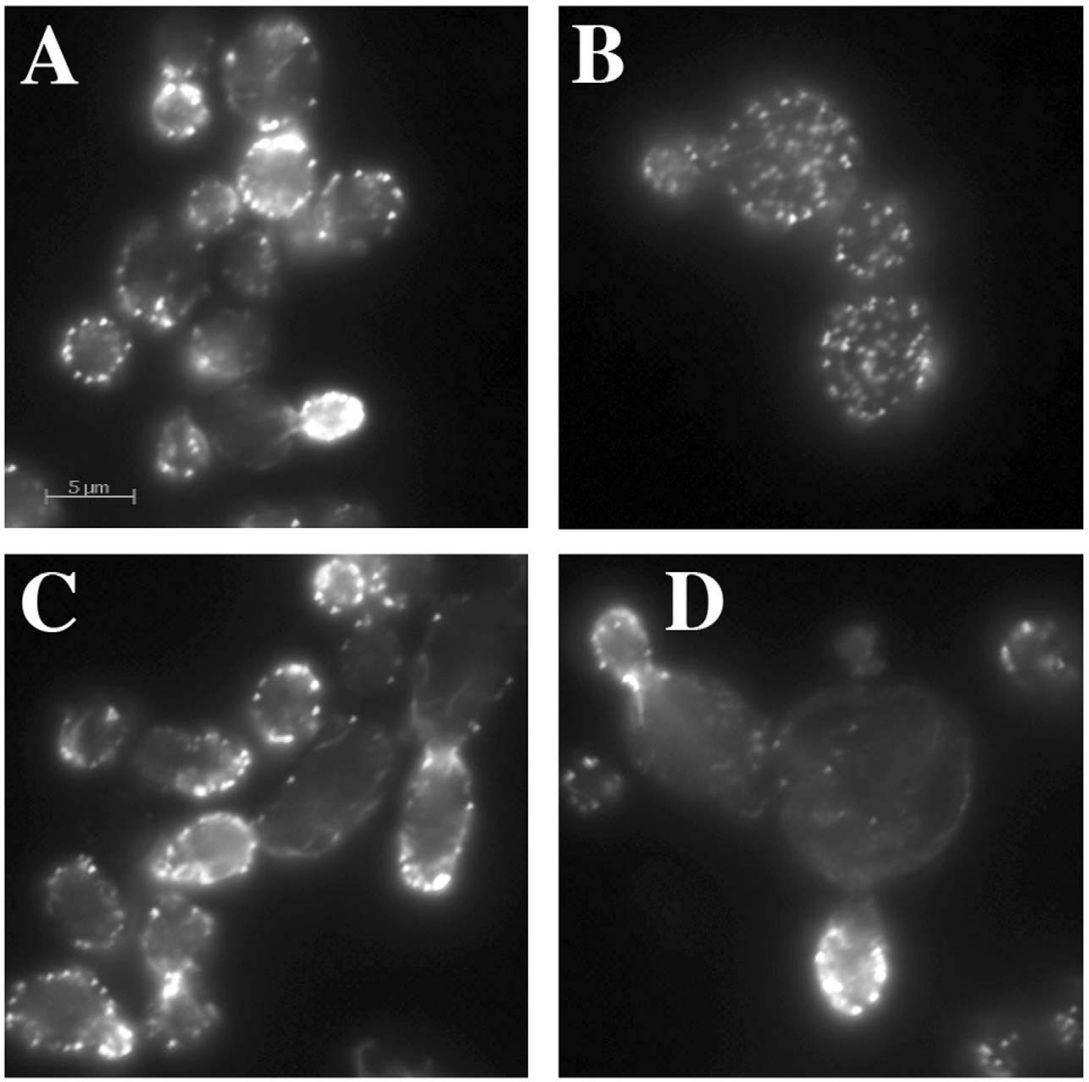
Actin staining of complex hemizygotes. *ACT1/act1Δ::Nat^R^* hemizygotes carrying additional hemizygous mutations for (A) *yal066wΔ::kan^R^* (control gene deletion), (B) *pfy1*Δ*::kan^R^*, (C) *rpn5*Δ*::kan^R^*, and (D) *tub1*Δ*::kan^R^* were fixed and stained with rhodamine phalloidin after growth at 30° in YPD medium.

A normal wild type cell typically has 4–5 actin cables that run along the long axis of the cell, terminating at the bud neck or extending into the bud. Actin cables are used by myosin motors to facilitate the polarized transport of materials into the bud. The other prominent actin structures are actin cortical patches, which in a wild type cell are polarized in the bud for most of the cell cycle and become polarized to the bud neck near the end of the cell cycle. Actin cortical patches are sites of endocytosis. For an example of normal actin organization, see “WT” in Figure 5C. Actin-specific phenotypes are relatively normal for most hemizygotes of the *act1* CHI set (data not shown). We had previously reported that the non-essential genes whose null alleles are CHI with actin displayed a surprising frequency of actin organization defects as haploids [7]. A comparable analysis cannot be performed with the essential gene null alleles that are CHI with actin, instead we examined the actin cytoskeleton in some of the complex hemizygotes. This analysis was complicated by the fairly severe actin organization defects in the *act1* hemizygote alone, which has abundant but depolarized actin cortical patches and poor actin cable formation and polarization (see Figure 3B). We did note some actin and morphological defects beyond those of the *act1* hemizygote (Figure 3A) in several complex hemizygotes, including those carrying *pfy1* (Figure 3B), *rpn5* (Figure 3C), and *tub1* (Figure 3D), while others had few, if any, additional defects. Lowering actin levels appears to affect actin cable formation more seriously suggesting that the patch F-actin nucleation machinery (the Arp2/3 complex) is less sensitive to low actin concentrations than the cable nucleation machinery (the formins). The larger, rounder cells of the *act1/ACT1^wt^ pfy1Δ/PFY1^wt^* complex hemizygote and extreme patch depolarization (Figure 3B) are reminiscent of various *pfy1* point mutants and represent a much less severe phenotype than that of barely viable *pfy1Δ* haploid cells [14]. Many of the complex hemizygotes with deletions of proteosome component genes showed an elongated cell and bud phenotype, an example of which is shown for *rpn5* in Figure 3C. The *ACT1/act1 tub1/TUB1* hemizygotes displayed severe and heterogeneous defects in actin organization and cell morphology. In many cases actin assembly was overly elaborate and completely polarized in the bud (Figure 3D) which is consistent with our previous observation that stabilization of filamentous actin is detrimental when actin is limiting [7].

### Functional enrichment in the essential genes that are CHI with *act1Δ*

As in our previous study [7], we noted that several genes whose null alleles are CHI with *act1Δ* express proteins that fall into common pathways or complexes. Hand testing of strains bearing null alleles for other (previously not hit) members of these pathways or complexes resulted in the identification of 12 additional CHI interactions with *act1Δ*. This observation alone argues that there is functional enrichment in the network. Some functional overlap was observed between this and our previous screens of the non-essential knock-outs. For example, here we found interactions with null alleles for two essential components of the Arp2/3 complex (*ARP3* and *ARC19*) and a null allele for the Arp2/3 activator *LAS17*. We also found an interaction with a null allele for one of the few (nearly) essential actin binding protein genes (yeast profilin; *PFY1*), and we found interactions with null alleles for two genes for the essential Tcp1p chaperone complex, which helps to fold actin and tubulin subunits; the previous screen saw interactions with null alleles for several components of the separate and non-essential prefoldin chaperone complex that also helps to fold both actin and tubulin subunits [15].

Our original CHI screen against the non-essential genes missed many cellular functions that are executed by essential genes. Although the screens against the non-essential knock-outs identified many genes involved in endocytic processes, most of the exocytic genes that had been frequently found in previous classical genetic screens with actin (e.g. suppression screens; [16]) were missed because these are almost entirely essential. Here we saw an interaction with the null allele for the late secretory vesicle small GTPase Sec4 and null alleles of genes for two components of the coatamer complex (*RET2* and *COP1*) that facilitate retrograde transport from the golgi to the ER. Also notable are the interactions with the tubulin cytoskeleton, in particular *tub1Δ* whose wild type allele encodes for alpha-tubulin, *tub4Δ* whose wild type allele codes for gamma-tubulin that nucleates microtubules at the spindle pole body, and *spc42Δ* whose wild type allele codes for a core component of the outer plaque of the spindle pole body. Since actin has been shown to be involved in nuclear positioning and segregation during mitosis ([17] and references cited within) the interactions with tubulin and spindle pole body genes may reflect catastrophic defects in nuclear positioning under conditions of limiting actin concentration. Statistical analysis does confirm that the network is enriched for genes annotated to the GO component term “cytoskeleton” with a p value = 6.62×10^−13^. This includes the genes: *PFY1*, *TUB1*, *LAS17*, *CCT4*, *TUB4*, *ARC19*, *SPC42*, *SEC4*, *CCT8*, and *ARP3* [18].

Most striking were the extensive interactions with null alleles of genes for the TFIID complex and the proteasome. Knock-out alleles of nearly all of the TAFs that make up the proteins of the TFIID complex were found to be CHI with *act1Δ*; statistical analysis for GO-term enrichment gave a p-value of 1×10^−14^ for the GO Biological Process term “transcriptional pre-initiation complex assembly” [19]. This could be the result of poor expression of actin and/or actin binding proteins, a hypothesis that is supported by CHI interactions between *act1Δ* and deletion alleles for three components of the RNA polymerase II complex (*RPB7*, *RPB3*, and *RPC10*). However, since actin has been found to be a core component of the RNA polymerase II pre-initiation complex [20] and to be involved in transcriptional elongation [21], these interactions may reflect a more direct role of actin in transcription. For example, stoichiometry suggests that actin in the RNA polymerase II complex is in the monomer form [19] and we have shown that the actin hemizygote is particularly limited for G-actin [7].

Particularly surprising are the extensive CHI interactions with null alleles for genes of the proteasome. Of the 33 genes that encode for all components of the 26S proteasome, we found that the knock-outs for 15 were CHI with *act1Δ*. The p-value for enrichment of the GO biological process term “ubiquitin-dependent protein catabolic process” is a remarkable 6.5×10^−13^. The genetic interaction between actin and proteasome genes could also be the result of indirect effects such as alterations in the protein levels of actin or key actin regulators. We have not performed an extensive analysis of the levels of all actin regulators but have looked at actin itself and its levels are unchanged in proteasome mutants. Alternatively, it could reflect a direct role of the proteasome in the regulation of the actin cytoskeleton or vice versa.

### Null alleles for subsets of proteasome component genes are CHI with *act1Δ*

**Figure 4:**
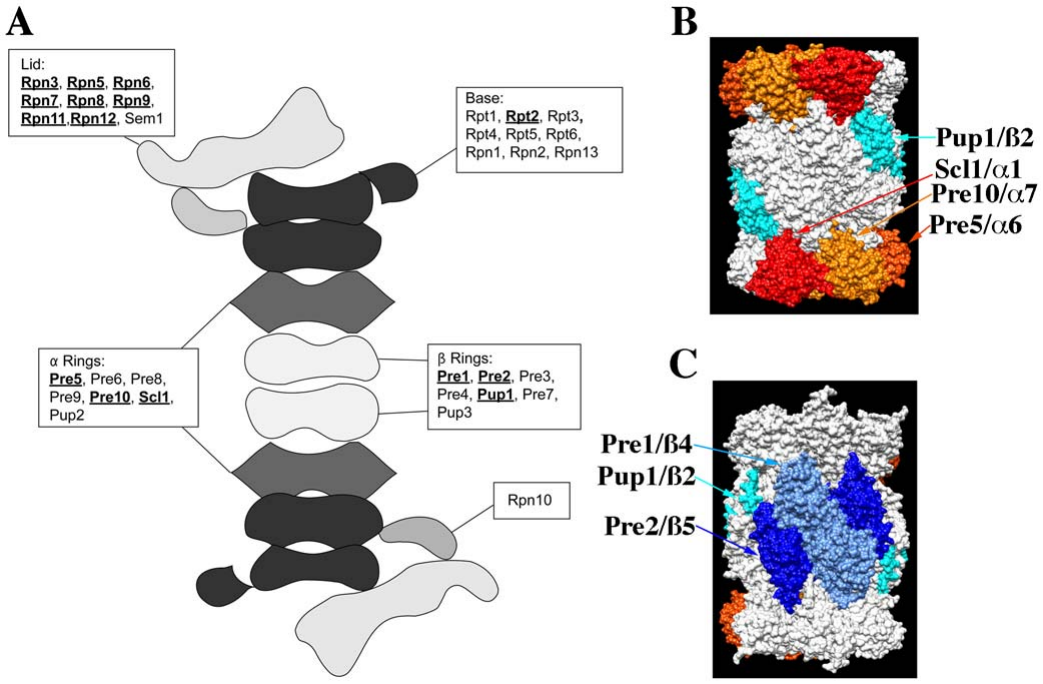
The structure of the proteasome and the locations of subunits whose null alleles are CHI with *act1Δ*. (A) Cartoon of the structure of the 26S, doubly capped proteasome showing the general locations of subunits whose null alleles are CHI (bold and underlined) or are not CHI with *act1Δ*; based on cryo-EM reconstructions of the 26S proteasome [47]. (B) and (C) Surface rendering of the 20S core proteasome X-ray structure (1RYP.pdb; [25]); panels are rotated 180° with respect to each other. Proteins whose null alleles are CHI with *act1Δ* have been rendered in color: red for Scl1p, gold for Pre10p, orange for Pre5p, teal for Pup1p, light blue for Pre1p, and dark blue for Pre2p. Rendering was performed with Chimera (http://www.cgl.ucsf.edu/chimera).

The proteasome is a large macro-molecular complex that executes much of the protein degradation activity of the eukaryotic cell (see Figure 4). It consists of a 20S core particle (CP) that is capped at one or both ends by a 19S regulatory particle (RP; see Figure 4A). The CP consists of two central, heptameric ß-rings composed of 7 different ß-subunits that are sandwiched between two α-rings similarly composed of 7 α-subunits. The CP contains the proteolytic sites while the RP is involved in substrate recognition and protein unfolding. The RP can be further subdivided into a lid complex that is coupled to the base complex by the Rpn10 protein [22]. Interestingly, nearly all proteasome components (with the exception of the α-3 subunit Pre9p and the cap coupling subunit Rpn10p) are essential and presumably required for proteasome activity, and yet not all knock-outs of proteasome component genes display a CHI interaction with *act1Δ* (see Figure 4A). Concerning the RP, we observed that null alleles for all genes of the lid complex components except *SEM1* were CHI with *act1Δ*. In contrast, a knock-out for only one gene (*RPT2*) of the base complex was CHI with *act1Δ* and a null allele for the Rpn10p linker was also not CHI with *act1Δ*. With respect to the 20S core, we observed that null alleles for only three of the alpha subunit genes (*PRE5*, *PRE10*, and *SCL1*) and three of the ß-subunit genes (*PUP1*, *PRE1*, and *PRE2*) were CHI with *act1Δ*. Note that three ß-subunit genes encode for the three proteolytic activities of the proteasome and two of the three are CHI with actin; *PUP1* encodes for the subunit with trypsin-like activity and *PRE2* encodes for the chymotryptic activity [23]–[24]. Interestingly, only the chymotryptic activity of the proteasome is essential [24].

The crystal structure of the proteasome 20S core indicates that each of the alpha and beta subunits occupies distinct locations within the two α– and ß-rings [25]. Figure 4B and 4C shows surface renderings of the structure of the 20S proteasome core with the CHI components colored and labeled. Strikingly, the α-ring CHI subunits are adjacent to each other and generally do not overlap with the ß-ring CHI subunits, such that α- and ß-subunits lie on opposite surfaces of the barrel-shaped core. The asymmetry of CHI interactions for proteasome subunit genes could reflect that these components are limiting and that reductions in their levels leads to defects in proteasome assembly. For example, the juxtaposition of CHI and non-CHI subunits in the 20S core particle may reflect the roles of co-templated interactions between subunits of the α- and ß-rings during the assembly of the intermediate, 20S CP ½ ring complex [26].

### Proteasome mutants have diverse defects in actin organization

To examine if there may be a functional connection between the proteasome and actin, we obtained several Ts^−^ alleles of proteasome component genes ([27] and gift of C. Boone) and examined their growth characteristics on both plate media and in liquid cultures after a shift to 37°C. We noted that the proteasome mutants varied in their extent of growth defects at 37°C. Interestingly, some proteasome mutants failed to form colonies at 37°C but appeared to grow relatively normally in liquid cultures at 37°C. Typically we have observed that strains with Ts^−^ alleles tend to behave similarly on both plate and in liquid cultures (with the exception of the *ACT1* hemizygote) and so the discrepancies observed for proteasome mutants may reflect something special about these genes or the proteasome in general. The growth behavior of the proteasome mutants is noted in Table 2.

**Figure 5:**
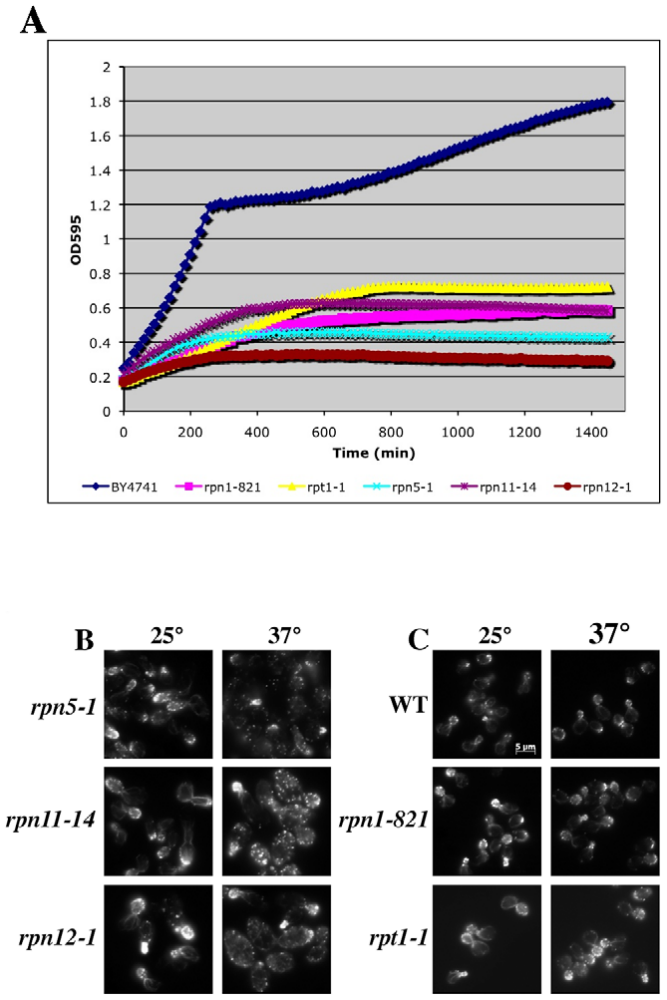
Growth and actin organization defects in conditional mutants of proteasome components. (A) Growth of strains carrying temperature sensitive alleles of proteasome genes were monitored and compared to the growth of a wild-type strain (BY4741) in a TECAN microplate reader at 37°C for 24 hr. (B and C) Strains carrying Ts^−^ alleles in proteasome component genes were grown to mid-log, shifted to 37°C for 2 hr, fixed, stained with rhodamine phalloidin and visualized by fluorescence microscopy. (B) Proteasome mutant strains with actin organization defects. (C) Proteasome mutant strains that do not have actin organization defects.

To examine the actin cytoskeleton in proteasome mutants, we stained log-phase cultures of proteasome mutant strains with rhodamine-phalloidin following a 2 hr shift to 37°C and examined actin organization by fluorescence microscopy. Interestingly, there was a wide range of phenotypes observed, from completely wild type actin organization to very severe defects in actin organization (see summary in Table 2 and examples of actin staining in Figure 5B). We scored a mutant as having severe defects when virtually all the cells in the culture were mutant: generally very large cells usually lacking cables and with numerous depolarized patches. Moderate defects indicate cultures in which most of the cells have morphology defects, generally large and sometimes hyper-elongated cells with excessive cables. Actin defects did not strictly correlate with whether the null allele for the proteasome mutant is CHI with actin, whether the subunit it encodes is in a particular sub-complex of the proteasome, or the extent of temperature sensitivity of the mutant strain. In Figure 5 we highlight the results from a set of mutants that have largely equivalent Ts^−^ phenotypes in liquid culture as shown by growth curves generated using a TECAN microplate reader (Figure 5A). This included alleles for three lid complex genes (*rpn5-1*, *rpn11-14*, and *rpn12-1*) and alleles for two base complex genes (*rpn1-821* and *rpt1-1*). As can be seen in Figure 5B, the three lid complex mutants have severe actin and cell morphology defects that are notable at permissive temperature (25°C) and get even worse at 37°C. At 25°C the cells are large, frequently elongated with elongated buds, the actin cortical patches are well polarized, and the actin cables are present and polarized. At 37°C, the cells became extremely large with depolarized cortical patches and if actin cables are present they are largely disorganized. This phenotype was typical for those proteasome mutants that display actin organization defects and although these strains are presumably arresting their growth due to defects in proteolysis, the actin defects alone are severe enough to possibly cause inviability. In stark contrast, the *rpn1-821* and *rpt1-1* mutant cells were completely normal for both cell morphology and actin organization at both 25°C and 37°C (Figure 5C). The kinetics of growth arrest at 37°C for all strains was similar and yet only some of the mutants arrest with actin defects. Although we do not know the reasons for this phenotypic variation, we suspect it may relate to differences in how the mutants affect proteasome assembly and function, in particular a possible actin specific function.

### The proteasome inhibitor MG132 elicits distinct defects in cell morphology and actin organization

**Figure 6:**
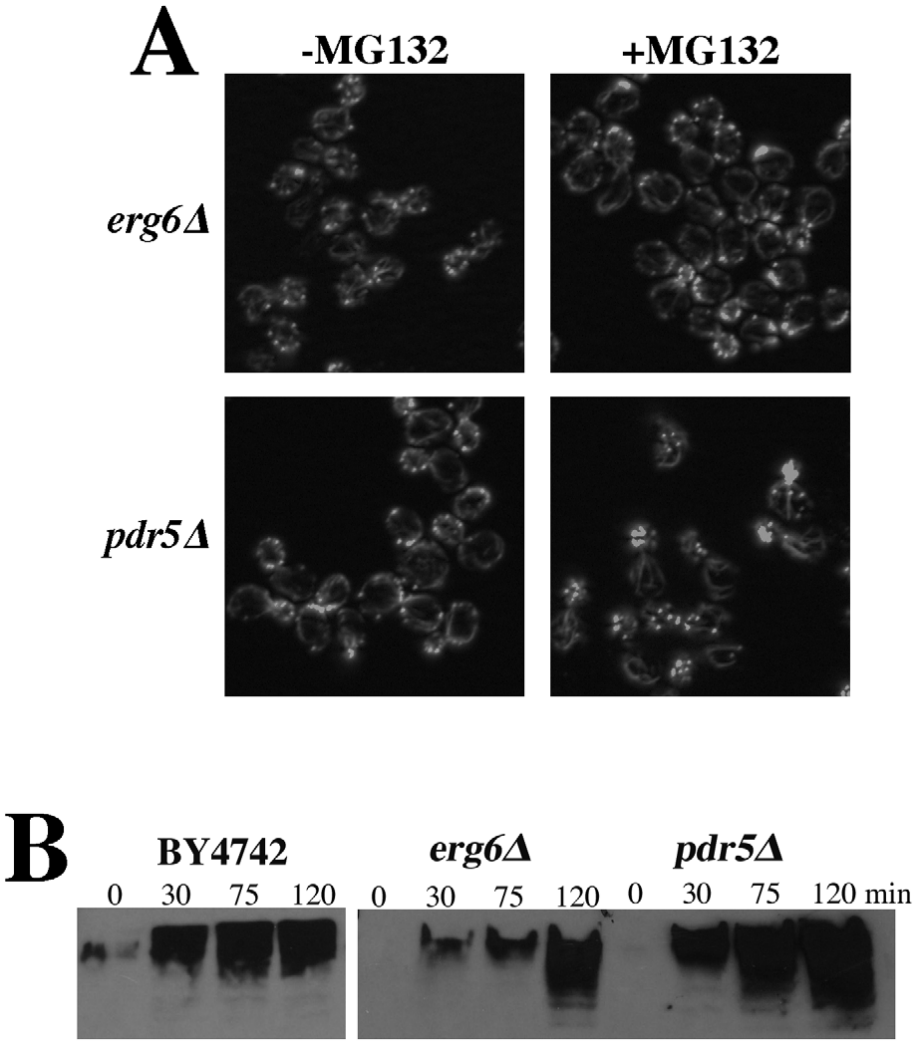
Cells treated with the proteasome inhibitor MG132 have normal actin cytoskeleton organization. (A) To facilitate drug permeability or accumulation, *erg6Δ* and *pdr5Δ* strains were grown to mid-log, treated with 100 µM MG132 for 2 hr, fixed, stained with rhodamine phalloidin and visualized by fluorescence microscopy. (B) Wild-type (BY4742), *erg6Δ*, and *pdr5Δ* cells were treated with 50 µM MG132 and protein samples were isolated 0, 30, 75, and 120 min after MG132 addition to the medium. Protein concentrations were determined by Bradford assays and ∼45 µg of protein were separated by SDS-PAGE and analyzed by Western blotting with an anti-ubiquitin antibody.

**Figure 7:**
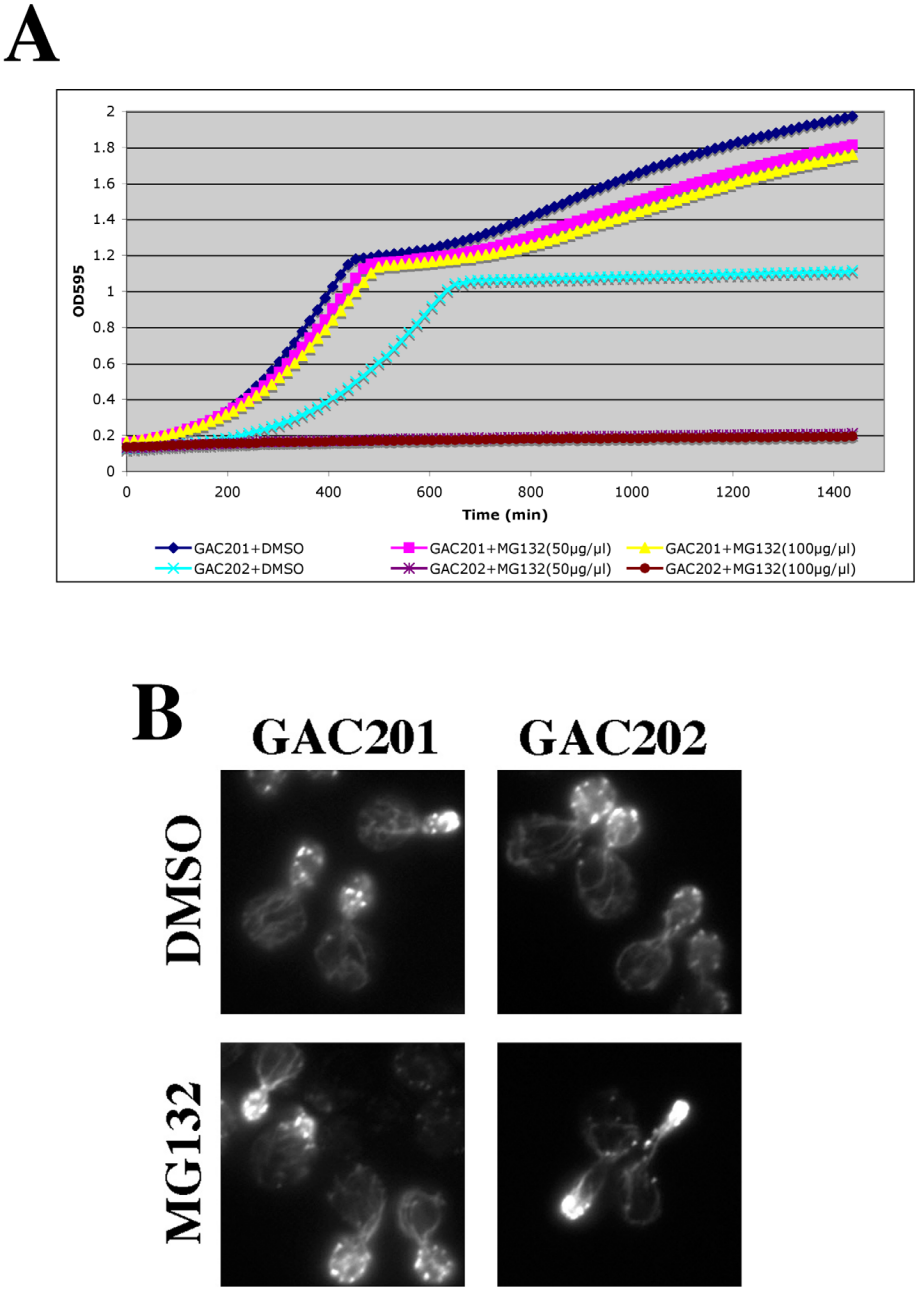
Complete inhibition of the proteasome induces a hypolarization phenotype. (A) A *pdr5Δ/PUP1^WT^/PRE3^WT^/PRE2^WT^* (GAC201) and a *pdr5Δ/pup1^T30A^/pre3^T20A^/PRE2^WT^* (GAC202) strain were grown into mid-log phase, treated with DMSO, 50 µM, or 100 µM MG132 and their growth was monitored in a TECAN microplate reader at 30°C. (B) The GAC201 and GAC202 strains were grown into mid-log phase, treated with 100 µM MG132 (or with a comparable amount of the DMSO solvent) for 2 hr, fixed with formaldehyde, stained with rhodamine-phalloidin and visualized by fluorescence microscopy.

The actin organization defects observed in the *rpn5*, *rpn11*, and *rpn12* Ts^−^ strains (and others) were expected to be attributable to loss of proteolysis activity of the proteasome complex. The lactocystin derivative MG132 is a commonly used proteasome inhibitor that covalently modifies the active site threonine of the chymotryptic center of the Pre2p subunit [25]–[26], [28]. Although MG132 specifically targets the chymotryptic activity of the proteasome, it significantly reduces the proteolytic activity of the other two ß-subunits as well [28]. This and related derivatives have been used extensively to study mammalian cell proteasome function but its utility in studying yeast proteasome function is limited by cell permeability issues. However, an ergosterol biosynthesis mutant (*erg6*) has been shown to increase cell permeability to a number of drugs, in particular an *erg6* mutation has been shown to render yeast cells sensitive to MG132 [29]. We grew an *erg6Δ* strain to mid-log, treated it +/−100 µM MG132 for 2 hr and stained the actin cytoskeleton with rhodamine-phalloidin. A comparable treatment of an *erg6* mutant strain had previously been show to cause an ∼80% inhibition of *in vivo* proteasome activity [29]. Despite an assumed comparable inhibition in our hands, we saw absolutely no effect of MG132 treatment on actin organization (Figure 6A). Deletion of the gene for the ABC, multi-drug transporter Pdr5p has also been shown to render yeast cells sensitive to 50 µM MG132 [30]. However, as similarly observed for the *erg6Δ* strain, treatment of a *pdr5Δ* strain with 100 µM MG132 for 2 hr had no effect on actin organization (Figure 6A). To confirm that the MG132 was in fact inhibiting the proteasome, we isolated proteins from cells treated with 50 µM MG132, separated the protein samples by SDS-PAGE and Western blotted with anti-ubiquitin antibodies (Figure 6B). Both the *erg6Δ* and *pdr5Δ* cells as well as the wild-type control accumulated ubiquitinated proteins during the 2 hr treatment with MG132. However, we noted that both *erg6Δ* and *pdr5Δ* strains continue to grow in the presence of 100 µM MG132 (see Figure 7A; GAC201 is a *pdr5Δ* strain) indicating that the residual activity of uninhibited proteasomes is sufficient to maintain cell growth and viability despite the accumulation of significantly high levels of ubiquitinated proteins.

It has been shown that yeast can survive without the trypsin-like activity of Pup1p and the caspase-like activity of Pre3p [23]. More recently a strain was developed in which the active site threonines of both Pup1p and Pre3p were mutated, rendering the strain completely reliant on the chymotrypsin activity of the Pre2p subunit. This strain also carries a *pdr5Δ* allele, rendering it completely sensitive to MG132 for cell growth and viability [31]. We confirmed the sensitivity of this strain to MG132 by performing TECAN growth curves (Figure 7A), note that the hyper-sensitized GAC202 strain arrests growth immediately following the addition of MG132 (Time 0). We treated this strain, and a control strain bearing wild type alleles of *PUP1* and *PRE3*, +/−100 µM MG132 for 2 hr followed by rhodamine-phalloidin staining to visualize the actin cytoskeleton (Figure 7B). Strikingly, the MG132 sensitive strain treated with MG132 displayed a high percentage of cells with F-actin accumulation at the tips of long and narrow buds. We quantified the percentages of cells with these hyper-elongated buds and found that in cultures of the MG132-sensitive strain grown in MG132, nearly 20% of the cells displayed this phenotype as compared to at most 3% in control cultures (see Table 3). Since the control strains carry plasmids encoding the wild type copies of *PUP1* and *PRE3*, we assume the low percentage of cells with elongated buds observed in the MG132-treated control strain are the result of plasmid loss. Interestingly, this elongated bud phenotype is distinct from that observed in proteasome subunit Ts^−^ strains (see Figure 5B). Elongated buds are observed in some Ts^−^ proteasome mutants but they tend to be broader, particularly in the neck region. The overwhelmingly common phenotype at 37°C, for proteasome mutants that do show actin defects, is large cells with a depolarized actin cytoskeleton. In contrast, the arrest phenotype observed in the MG132 hypersensitive strain is actin hyper-polarization and is more reminiscent of a cell cycle defect, likely reflecting the well-established role of the proteasome in degradation of cell cycle regulators [32].

### Both the 19S regulatory and the 20S catalytic particles of the proteasome precipitate in association with actin filaments

**Figure 8:**
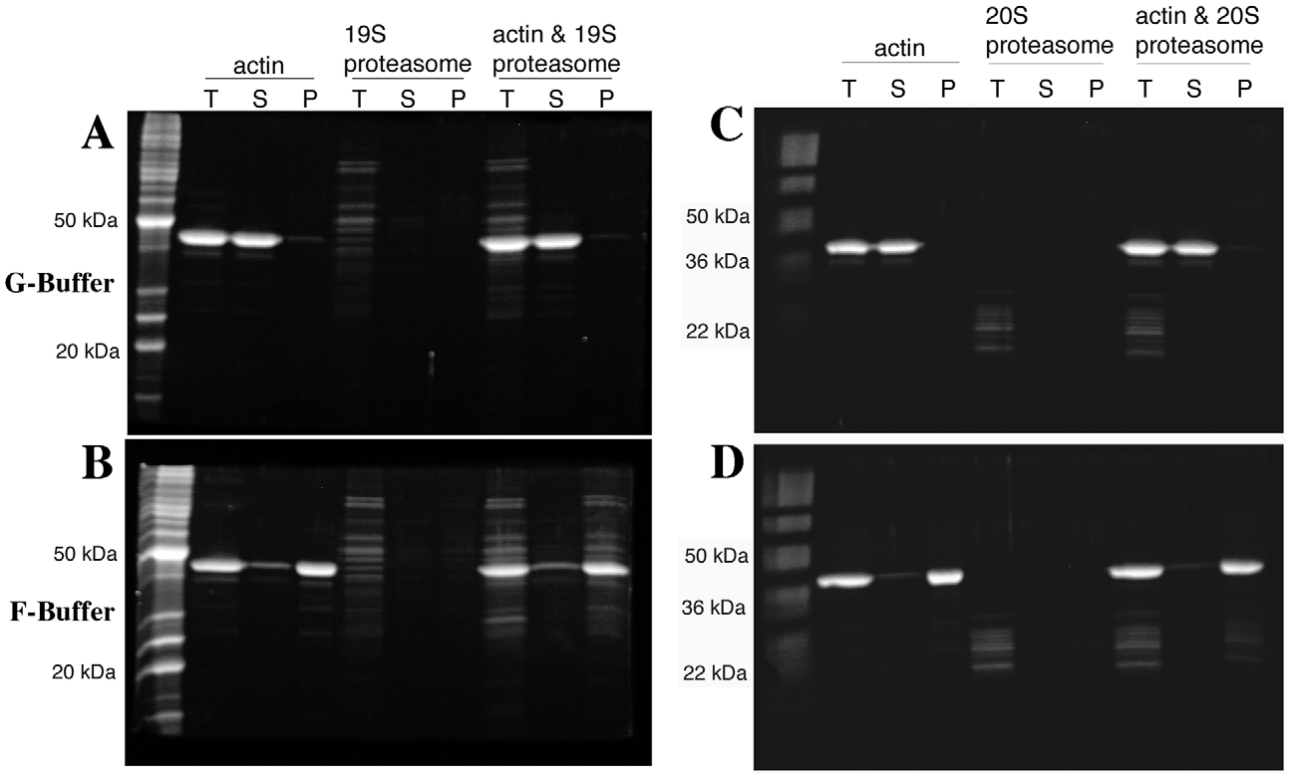
Both the proteasome 19S regulatory particle and 20S core particle associate with actin filaments. 4 µM yeast actin and either ∼65 nM 19S proteasome regulatory particle (A and B) or 50–100 nM 20S proteasome core particle (C and D) were incubated in G-buffer (A and C) or in F-buffer (C and D), centrifuged at 190,000×g and volume-corrected samples were taken from the supernatant (S) or pellet (P) phases. T are the fractions taken prior to centrifugation. All fractions were separated by SDS-PAGE and stained with SYPRO Ruby (Bio-Rad, Hercules CA).

The diversity of actin organization defects observed in some proteasome mutants suggested that perhaps the proteasome could be regulating actin via a direct interaction. To test this possibility, we purified assembly competent actin and the 19S proteasome RP from yeast. Actin filament pelleting assays were used to assay for 19S RP association specifically with actin filaments. When the actin is kept in the monomeric form by incubating in low salt G-buffer and submitted to high-speed centrifugation at 190,000× g, nearly all of the actin remains in the supernatant phase (Figure 8A). When the actin was incubated in the presence of ∼65 nM yeast 19S RP, the actin remained in the supernatant in G-buffer and none of the 19S RP was found in the pellet fraction (Figure 8A). However, when actin polymerization was stimulated with salt and magnesium in F-buffer, most of the actin is found in the pellet phase (see Figure 8B) and most of the 19S RP proteins pellet with the actin filaments. Note that the 19S RP is not found in the supernatant fraction as this fraction is taken from the very top of the reaction; we have sampled down the column of F-buffer centrifugation reactions and consistently find that the 19S RP migrates to a fraction above the pellet (data not shown). To confirm the apparent association of the 19S RP with actin filaments, we analyzed both the 19S preparation and the F-actin plus 19S pellet by mass-spectrometry, specifically reverse phase chromatography in line with tandem MS using an LTQ Orbitrap XL. The 19S prep was confirmed to contain multiple high confidence peptides for all of the lid and base components, for the Rpn10p linker, and for the Rpn14p proteasome assembly factor. In the F-actin plus 19S pellet a large number of actin peptides were identified, multiple peptides for all 9 base components, multiple peptides for 7 of 8 lid components but only 1 peptide for lid component Rpn5p (data not shown). It is likely that the large concentration of actin peptides in the pellet fraction obscured the identification of some 19S RP peptides. Regardless, the mass-spectrometry results provide strong support for the physical association of the full 19S RP with actin filaments in our pelleting assays.

To examine for a potential association between the 20S core proteasome and F-actin, pelleting assays were repeated with preparations of 20S core purified from a strain expressing Flag-tagged Pre1p [33]. As was observed for the 19S RP (Figure 8A), when the actin was incubated in the presence of 50–100 nM yeast 20S proteasome, the actin remains in the supernatant fraction (Figure 8C). When actin polymerization was stimulated in F-buffer, most of the 20S CP proteins pelleted with the actin filaments (see Figure 8D). Sampling of the entire fractionation consistently showed that the 20S proteasome, like the 19S RP, migrated to a fraction above the pellet (data not shown). The strong association of the 19S RP with F-actin suggests that 19S RP contamination in the 20S prep could be bridging the F-actin/20S association. To determine if 19S RP is present in the 20S preps, we performed the mass-spec analysis of the 20S preps and did find that the 20S preparation is contaminated with 19S RP proteins. However, the converse was not true; no 20S contamination was found in the 19S preparations (data not shown).

## Discussion

Although DNA replication and repair occur with impressive fidelity, the sheer amount of information that must be copied ensures that every time a genome is replicated, heritable mistakes are made. Over time, these errors and larger scale changes in chromosomes such as transposon movement, repeat expansion and contraction, and chromosome rearrangements have led to significant genetic diversity in populations. These genetic alterations have resulted in complex heterozygosity at many loci that are re-assorted every generation, resulting in a moving target of complex genetic interactions whose influence on phenotype is difficult to ascertain. Many, but not all, mutations are silent but it has been estimated from deep sequencing of 179 human individuals, that we all inherit 250–300 loss-of-functional alleles. Cancer cells represent perhaps the most extreme case of genetic diversity, as they frequently lose whole chromosomes or whole sections of chromosomes, resulting in the potential for thousands of complex haploinsufficient interactions. In our work we have been attempting to model CHI interactions in a simple eukaryote and specifically using the single essential actin gene, which plays critical roles in a large number of cellular functions. Our results confirm that there is a tremendous potential for deleterious CHI interactions in the genome. However, actin is so centrally important, we cannot say that all genes will be so genetically promiscuous.

Genetic interaction analysis has been used extensively in yeast to attempt to identify functionally related genes, the well supported assumption of course being that a binary gene interaction suggests relatedness. Such screens have led to a fairly anecdotal lore about what a particular type of interaction means, e.g. suppressor screens tend to identify gene products that physically interact or at least operate in the same pathway. The tremendous amount of synthetic lethal data generated by the Boone laboratory has pushed the ability to formalize rules about synthetic lethal interactions and how patterns of such interactions can be accurately used to determine function in same or parallel pathways [4]–[5]. Although very few CHI screens have been performed at this time, we expect that many of the lessons learned from synthetic lethality will hold true.

It is not surprising that a null allele for actin would have such a large number of CHI interactions; the actin cytoskeleton is centrally important to many cellular functions. Further, actin can exist in many highly dynamic forms that are regulated in complex spatio-temporal ways by a large number of associated proteins. Interpretation of any single CHI interaction will need to take into account these complexities. One thing that is clear is that CHI screens, like synthetic lethal screens, identify groups of functionally related genes and, with respect to actin, we can conclude that some very important cellular processes are hyper-sensitive to reductions in actin expression.

Since actin itself is haploinsufficient, we may have expected our screen to be overwhelmingly biased toward other genes that also display simple haploinsufficiency. However, this does not appear to be the case, thus bolstering the argument that, like other genetic interactions, a CHI interaction reflects functional specificity. Another possible explanation for CHI interactions could be cumulative reduced biosynthetic capacity [34], but our original CHI screen against the non-essential genes was not overly enriched for genes involved in biosynthesis, with the exception of the ribosome. The CHI screen against the essential genes presented here did identify a number of genes whose products are components of the proteasome and the core transcriptional apparatus, but we could not detect significant changes in the levels of actin, or the actin binding proteins Aip1p and cofilin in strains hemizygous for *RPN5*, *RPB3*, *RPC10*, *TAF5*, or *RPS5* (data not shown) suggesting these interactions may reflect true functional connections.

A common interpretation of synthetic lethal interactions between null alleles is that the two genes operate in parallel pathways that impinge upon an essential function [4]. A fundamental difference of a CHI interaction is that since activity from both genes is reduced by at most 50% and not 100%, the pathways the genes function in are not blocked entirely. Therefore, we might expect that a CHI interaction may frequently be indicative that the two genes/gene products function within the same pathway or structure; that pathway function is compromised by loss of flux due to constrictions at two steps. Given the striking enrichment for proteasome genes and the novelty of a proteasome-actin connection, we chose to investigate a potential functional connection between the actin cytoskeleton and the proteasome.

Analysis of Ts^−^ proteasome mutants showed that many, but not all, have severe defects in actin organization. In some cases these defects are so severe that this aspect of the phenotype alone might be expected to cause cell death. The data suggest, from two perspectives that the actin phenotypes cannot be purely explained by losses in proteasome proteolytic activity. First, treatment of sensitized cells with the proteasome proteolytic activity inhibitor MG132 resulted in very distinct defects in cell polarity and actin polarization that differs from that observed in Ts^−^ proteasome mutants. Second, several Ts^−^ proteasome mutants that cease growth entirely at 37°C arrest without any actin organization defects. We hypothesize that the phenotypic diversity observed in the proteasome mutants likely reflects differential effects of the alleles on multiple proteasome activities. For example, loss of proteolytic activity may indirectly affect actin and cell polarity. However, we hypothesize there is a second function that is evident when there are defects in a direct interaction between actin and proteasomes.

This hypothesis is supported by actin filament pelleting assays suggesting a direct physical interaction between the proteasome and actin filaments, although we cannot rule out possible non-specific interactions between two very large complexes. However, we failed to see pelleting of a BSA control with actin filaments indicating that actin is not merely “sticky” under these assay conditions (data not shown). Nonetheless, the data suggest that there are F-actin binding sites on both the 19S RP and the 20S CP. However, since mass-spectrometry identified 19S proteins in the 20S preparations, we cannot preclude the possibility that the 19S RP was bridging an interaction between F-actin and the 20S core in our assays. In either case, either double-capped 26S proteasome, or the 20S CP could be a bivalent actin filament cross-linking protein. This model agrees well with EM images showing a strikingly ladder-like cross-linking of rabbit muscle actin filaments by rabbit reticulocyte proteasomes [35]. These data suggest that the actin-proteasome interaction is conserved, which is supported by co-localization of proteasomes with the actin cytoskeleton in many different cell types including *Xenopus* oocytes [36], epithelial cells and fibroblasts [35], [37], myoblasts [38], and in the sarcomeres of muscle cells [37], [39]–[40].

One curious aspect of our proteasome data is that not all proteasome genes are CHI with actin and not all Ts^−^ alleles in proteasome genes cause actin organization defects. In our previous work on CHI interactions between actin and the non-essential gene knock-outs [7], we uncovered functional divergence between ribosomal paralog genes for actin-related defects. It has been shown that proteasomes with an alternative composition can be assembled in certain genetic backgrounds [41]. However, we do not believe that our data suggest the existence of a proteasome of alternative composition that has actin-specific functions. Instead, given that proteasome assembly has been shown to occur in a series of ordered steps [26], , we hypothesize that subunit limitation in hemizygous strains or mutation of certain proteasome subunits leads to the accumulation of assembly intermediates that selectively affect actin-specific functions of the proteasome. In regards to the phenotypic differences between Ts^−^ mutants of proteasome component genes, this likely reflects differences in the defects caused by the alleles. For example, some Ts^−^ mutants may affect function without affecting structure while others may affect both function and structure. Given our results with MG132 and actin organization, we hypothesize that proteasome structure is required for proper actin organization but catalytic activity may not be required for proper actin organization.

In summary, our results clearly prove the utility of CHI screening for making novel connections between cellular subsystems, for predicting gene function, and for mapping the landscape of binary gene interactions that are likely to be relevant to system collapse in more complex organisms including multigenic influences in human genetic disorders. In particular, extensive CHI interactions between actin and proteasome genes predicted a novel role for the proteasome in affecting actin cytoskeleton organization.

## Materials and Methods

### Complex haploinsufficiency screens

Complex haploinsufficiency screening was carried out as described previously [7], with the following changes: the EuroScarf (http://web.uni-frankfurt.de/fb15/mikro/euroscarf/) collection of heterozygous diploid strains carrying essential gene deletions was first pinned in 384-colony format to four YPD+G418 plates, incubated for two days at 25–30°, then transferred to sporulation plates. After 7–12 days at 25°, sporulation of randomly selected strains was confirmed (sporulation frequencies were generally between 1–5%) and these sporulated strains were mated on YPD to the *act1Δ* query strain BHY336, which contains *ACT1* on a *URA3*-based, centromere plasmid. Subsequent steps of diploid selection and CHI analysis were as described [7]. For some screens, the 384-colony plates were converted to 1536-colony format, equivalent to four-fold coverage per plate. Hand retesting was also as described previously, but with the additional step of first sporulating the heterozygous diploid strains prior to mating to BHY336.

### Growth curve analysis

For TECAN (Infinite F200)-based growth curves of CHI interacting strains, we picked colonies directly from diploid selection plates into liquid SC^msg^+G418+Nat+/−FOA and followed growth at 30° for ∼48 hours. Data were imported into Microsoft Excel for analysis and generation of growth curves. For TECAN (Infinite F200)-based growth curves of proteasome mutants strains, 25°C cultures in YPD medium at ∼2×10^7^ cells/ml were placed into wells of microtiter dishes and shifted to 37°C in the TECAN incubator/reader and cell density was monitored for 24 hr.

### F-actin staining

Rhodamine-phalloidin staining of actin was as described previously [43]. For rhodamine-phalloidin staining of complex hemizygotes, the haploid *act1Δ* query covered with the *URA3*-marked *ACT1^wt^* plasmid was mated to sporulations of the essential heterozygous diploid strains, the double hemizygotes were selected on medium containing NAT and G418, and plasmid loss was subsequently selected on 5-FOA medium. The resulting double hemizygotes were grown to mid-log in YDP medium and stained with rhodamine-phalloidin.

### MG132 treatments

Yeast strains were grown to mid-log phase (∼2×10^7^ cells/ml) and MG132 (A.G. Scientific Inc, San Diego, CA) dissolved in DMSO at 50 mM was added to a final concentration of 100 µM; an equal volume of DMSO was added to control cultures. The cells were incubated for 2 hr at 30°C, fixed in formaldehyde, and stained with rhodamine-phalloidin (see above).

### Detection of ubiquitinated proteins

Strains BY4742, *erg6Δ* and *pdr5Δ* were grown to mid log phase at 30°C and then treated with 50 µM MG132 (diluted from a 50 mM stock solution in DMSO) for 2 hours. 9 ml samples were harvested at time 0 min (before MG132 addition) and 30 min, 75 min and 120 min after MG132 addition. The cells were pelleted, the pellets were flash-frozen on dry ice and stored at −80°C. The pellets were thawed in 70 µl TBS (150 mM NaCl, 50 mM Tris-HCl, pH 7.4) supplemented with 1 mM PMSF (phenylmethanesulfonylfluoride) plus a 1∶250 dilution of Calbiochem Protease Inhibitor Cocktail IV (Calbiochem, La Jolla, CA) and lysed by vortexing with glass beads at 4°C. The protein concentrations of the cell lysates were determined by Bradford assay. The samples were diluted in an equal volume of urea-supplemented 2× sample buffer (50 mM Tris-HCl, pH 6.8, 8 M urea, 5% SDS, 1 mM EDTA, plus a 1∶20 dilution of a 1% bromophenol blue stock and a 1∶20 dilution of β-mercaptethanol), heated to 95°C for 5 min and ∼45 µg of total protein per well were separated by SDS-PAGE followed by transfer to nitrocellulose. Poly-ubiquitinated species were detected by Western blotting with anti-ubiquitin antibody clone Ubi-1 (Millipore, Billerica, MA) diluted 1∶500 in TBST (TBS plus 0.05% Tween 20) plus 5% dehydrated milk, followed by incubation in a secondary, HRP-conjugated goat anti-mouse IgG antibody (Sigma-Aldrich, St. Louis, MO) diluted 1∶1000 in TBST plus milk and detection with Supersignal (Thermo Scientific, Rockford, IL).

### Actin purification

Yeast actin was purified by a modified [44] DNaseI affinity purification procedure [45]. Briefly, 100 mg DNaseI (Roche Diagnostics, Indianapolis, IN) was coupled to 3 g of swelled Sepharose 4B (Sigma-Aldrich, St. Louis, MO). The coupled beads were packed into two disposable polypropylene columns of 5 ml maximum capacity (PIERCE/ThermoScientific, Rockford, IL). Each column was washed with 25 ml 0.2 M NH_4_Cl in G-buffer (10 mM Tris, pH 7.5, 0.2 mM CaCl_2_, 0.5 mM ATP and 0.2 mM DTT) followed by 25 ml G-buffer with 0.1 mM phenylmethylsulfonyl fluoride (PMSF). ∼100 g of yeast pellet (Red Star Yeast from an ∼400 g brick) were thawed in ∼100 ml G-buffer with 0.1 mM PMSF plus Calbiochem protease inhibitor cocktail IV diluted 1∶500. The cells were passed 8 times through a micro-fluidizer (Microfluidics, Model 110 L, Newton, MA). The lysate was clarified in a Beckman JA-20 rotor at 12,000 rpm for 30 min at 4°C and then in a Beckman Ti70 rotor at 50,000 rpm for 50 min at 4°C, followed by filtration through common coffee filters. The filtered supernatant was split between the two columns and loaded at a flow rate of 1–2 ml/min. Each column was washed with 25 ml of 10% deionized formamide in G-buffer, 25 ml of 0.2 M NH_4_Cl in G-buffer and 25 ml of G-buffer. The actin was eluted with 50% deionized formamide in G-buffer. The protein was dialyzed overnight in dialysis tubing (diameter 11.5 mm) with a molecular weight cut-off of 3,500 Da (Spectrum Laboratories, Rancho Dominiguez, CA) against 2 liters of G-buffer (25 µM ATP). The actin was further purified by FPLC on a 1 ml MonoQ column (Pharmacia Biotech, Piscataway NJ), washed with 10 ml of G-buffer at a flow rate of 0.5 ml/min, and then eluted with a linear, 0–300 mM KCl gradient in G-buffer. The actin eluted above 200 mM KCl; peak fractions (as estimated by the chromatograph and verified by SDS-PAGE gel) were pooled, distributed in 11×34 mm Beckman ultracentrifuge tubes (1 ml per tube) and polymerized in F-buffer (G-buffer+ATP to 1 mM+1 mM EGTA+4 mM MgCl_2_) for 20 min at room temperature. The KCl concentration was then brought up to 600 mM and the samples were incubated for 1 h at room temperature. Polymerized actin was pelleted by ultracentrifugation in a Beckman TLA100.2 rotor at 90,000 rpm for 30 min. Pellets were re-suspended in G-buffer to 1–2 mg/ml (as estimated by PAGE), incubated on ice for 2–4 hours and dialyzed overnight in 1 liter G-buffer. Protein concentration was determined by Bradford assay.

### Purification of yeast 19S and 20S proteasome components

Yeast 20S core proteasomal subunits were purified by an affinity purification procedure [33] as follows. ∼10 liters of strain RJD1144 [33] expressing flag-tagged Pre1p were grown overnight in synthetic medium, harvested, resuspended in 1 pellet volume lysis buffer (50 mM Tris, pH 7.5, 150 mM NaCl, 5 mM MgCl_2_), and frozen at −20°C. The cells were thawed in a room temperature water bath and then passed through a microfluidizer (Microfluidics, Model 110 L, Newton, MA) 10 times. The lysate was clarified in a Beckman JA-20 rotor at 17,000 rpm for 20 min and loaded onto a column packed with 1 ml anti-Flag M2 agarose beads (Sigma, St. Louis, MO). The column was washed with 50 ml lysis buffer+0.2% Triton, followed by two 50 ml washes with lysis buffer. The protein was eluted with elution buffer [25 mM Tris, pH 7.5, 150 mM NaCl, 50 mM MgCl_2_, 100 µg/ml FLAG peptide (Sigma, St. Louis, MO)], four 3 ml elutions were collected, the column was washed three times with 3 ml elution buffer minus the peptide and these fractions were collected as well. Protein concentrations were measured by Bradford assay, the fractions were pooled and the sample was dialyzed in dialysis tubing (diameter 11.5 mm) with a molecular weight cut-off of 3,500 Da (Spectrum Laboratories, Rancho Dominiguez, CA) against 1 liter G-buffer. The sample was then concentrated in a Vivaspin 15R concentrator (Sartorius Stedim Biotech, Bohemia, NY) and protein concentration was measured by Bradford assay.

For purification of the 19S proteasomal cap the same procedure was followed using strain RJD1171 (Table 1, [33]), which expresses flag-tagged Rpt1p.

**Table 1:** Strains used in this study.

Strain	Genotype	Source
BHY336	*MATα*, *his3Δ1*, *leu2Δ0*, *ura3Δ0*, *mfa1Δ::P_MFA1_-Sp_his5^+^* [pKFW29]	[7]
TSQ1340	*MATα*, *pre5-ph*::*Nat^R^*, *can1Δ*::*STE2^pr^-Sp_his5*, *lyp1Δ*	[27]
TSQ1349	*MATα*, *pup3-ph*::*Nat^R^*, *can1Δ*::*STE2^pr^-Sp_his5*, *lyp1Δ*	[27]
TSQ1350	*MATα*, *pre6-ph*::*Nat^R^*, *can1Δ*::*STE2^pr^-Sp_his5*, *lyp1Δ*	[27]
TSQ1354	*MATα*, *pre8-ph*::*Nat^R^*, *can1Δ*::*STE2^pr^-Sp_his5*, *lyp1Δ*	[27]
TSQ1355	*MATα*, *pre4-ph*::*Nat^R^*, *can1Δ*::*STE2^pr^-Sp_his5*, *lyp1Δ*	[27]
TSQ1371	*MATα*, *pup2-ph*::*Nat^R^*, *can1Δ*::*STE2^pr^-Sp_his5*, *lyp1Δ*	[27]
TSQ255	*MATα*, *rpn1-821*::*Nat^R^*, *can1Δ*::*STE2^pr^-Sp_his5*, *lyp1Δ*::*STE3^pr^-LEU2*	C. Boone
TSA255	*MAT* **a**, *rpn1-821*::*Kan^R^*, *his3Δ1*, *leu2Δ0*, *ura3Δ0*, *met15Δ0*	C. Boone
TSQ260	*MATα*, *rpt4-150*::*Nat^R^*, *can1Δ*::*STE2^pr^-Sp_his5*, *lyp1Δ*::*STE3^pr^-LEU2*	C. Boone
TSQ785	*MATα*, *rpt3-1*::*Nat^R^*, *can1Δ*::*STE2^pr^-Sp_his5*, *lyp1Δ*::*STE3^pr^-LEU2*	C. Boone
TSQ910	*MATα*, *rpt6-20*::*Nat^R^*, *can1Δ*::*STE2^pr^-Sp_his5*, *lyp1Δ*::*STE3^pr^-LEU2*	C. Boone
TSQ1090	*MATα*, *rpt1-1*::*Nat^R^*, *can1Δ*::*STE2^pr^-Sp_his5*, *lyp1Δ*::*STE3^pr^-LEU2*	C. Boone
TSQ1207	*MATα*, *rpt6-1*::*Nat^R^*, *can1Δ*::*STE2^pr^-Sp_his5*, *lyp1Δ*::*STE3^pr^-LEU2*	C. Boone
TSQ1263	*MATα*, *rpt4-145*::*Nat^R^*, *can1Δ*::*STE2^pr^-Sp_his5*, *lyp1Δ*::*STE3^pr^-LEU2*	C. Boone
TSQ405	*MATα*, *rpn12-1*::*Nat^R^*, *can1Δ*::*STE2^pr^-Sp_his5*, *lyp1Δ*::*STE3^pr^-LEU2*	C. Boone
TSQ521	*MATα*, *rpn11-8*::*Nat^R^*, *can1Δ*::*STE2^pr^-Sp_his5*, *lyp1Δ*::*STE3^pr^-LEU2*	C. Boone
TSQ534	*MATα*, *rpn11-14*::*Nat^R^*, *can1Δ*::*STE2^pr^-Sp_his5*, *lyp1Δ*::*STE3^pr^-LEU2*	C. Boone
TSQ1037	*MATα*, *rpn6-1*::*Nat^R^*, *can1Δ*::*STE2^pr^-Sp_his5*, *lyp1Δ*::*STE3^pr^-LEU2*	C. Boone
TSQ1235	*MATα*, *rpn5-1*::*Nat^R^*, *can1Δ*::*STE2^pr^-Sp_his5*, *lyp1Δ*::*STE3^pr^-LEU2*	C. Boone
RJD1144	*MAT* **a**, *his3Δ200*, *leu2-3, 112*, *lys2-801*, *trp1Δ63*, *ura3-52*, *PRE1^FH^*:: *URA3, pre1Δ*	[33]
RJD1171	*MAT* **a**, *his3Δ200*, *leu2-3*, *112*, *lys2-801*, *trp1Δ63*, *ura3-52*, *RPT1^FH^*:: *URA3, rpt1Δ*	[33]
BY4742	*MATα*, *his3Δ1,leu2Δ0*, *ura3Δ0*, *lys2Δ0*	EuroScarf
*erg6Δ*	*MAT* **a**, *his3Δ1,leu2Δ0*, *ura3Δ0*, *met15Δ0*, *erg6Δ0::G418^r^*	EuroScarf
*pdr5Δ*	*MAT* **a**, *his3Δ1,leu2Δ0*, *ura3Δ0*, *met15Δ0*, *pdr5Δ0::G418^r^*	EuroScarf
GAC201	*MATα*, *his3-11*, *leu2-3,112*, *ura3-52*, *lys2-801*, *trp1-1*, *pdr5Δ::KanMX6*, *pre3Δ2::HIS3*, *pup1Δ::leu2-HIS3*, [pRS317-*PUP1*], [YCplac-*PRE3*], gal^−^	[33]
GAC202	*MATα*, *his3-11*, *leu2-3,112*, *ura3-52*, *lys2-801*, *trp1-1*, *pdr5Δ::KanMX6*, *pre3Δ2::HIS3*, *pup1Δ::leu2-HIS3*, [pRS317-*pup1^T30A^*], [YCplac-*pre3^T20A^*], gal^−^	[33]

**Table 2:** The actin phenotypes of conditional proteasome mutants.

Complex	Gene	Allele	CHI with actin	Plate Growth	Liquid Growth	Actin phenotype
**Lid**	*RPN5*	*rpn5-1*	yes	No growth at 37°	Ts^−^	severe
	*RPN6*	*rpn6-1*	yes	No growth at 37°	Ts^+^	wild type
	*RPN11*	*rpn11-8*	yes	No growth at 37°	Ts^−^	severe
		*rpn11-14*		No growth at 37°	Ts^−^	severe
	*RPN12*	*rpn12-1*	yes	No growth at 37°	Ts^−^	severe
**Base**	*RPN1*	*rpn1-821*	no	No growth at 37°	Ts^−^	wild type
	*RPT1*	*rpt1-1*	no	Slow growth at 37°	Ts^−^	wild type
	*RPT3*	*rpt3-1*	no	No growth at 37°	Ts^+^	wild type
	*RPT4*	*rpt4-145*	no	No growth at 37°	Ts^−^	moderate
		*rpt4-150*		Slow growth at 37°	Ts^−^	moderate
	*RPT6*	*rpt6-1*	no	No growth at 37°	Ts^+^	wild type
		*rpt6-20*		No growth at 37°	Ts^+^	wild type
**α ring**	*PRE5*	*pre5-ph*	yes	Slightly slow growth at 37°	Ts^+^	wild type
	*PRE6*	*pre6-ph*	no	Slow growth at 25° and 37°	Ts^+^	wild type
	*PRE8*	*pre8-ph*	no	Slow growth at 37°	Ts^+^	severe
	*PUP2*	*pup2-ph*	no	Slow growth at 37°	Ts^+^	moderate
**β ring**	*PRE4*	*pre4-ph*	no	No growth at 37°	Ts^+^	wild type
	*PUP3*	*pup3-ph*	no	Slightly slow growth at 37°	Ts^+^	wild type

**Table 3:** Percentages of morphologically aberrant cells in MG132-treated cultures.

	GAC201+DMSO	GAC201+MG132	GAC202+DMSO	GAC202+MG132
**Un-budded**	23.2%	17.0%	27.1%	31.1%
**Normal Buds**	75.0%	80.3%	71.2%	48.4%
**Elongated Buds**	1.8%	2.7%	1.1%	**19.6%**
**Large Un-polarized**	0.0%	0.0%	0.5%	0.9%
**N = **	340	223	365	219

### F-actin pelleting assays

To evaluate F-actin-proteasome interactions, 4 µM yeast actin was left for 1 h at room temperature to reach equilibrium in G- (5 mM HEPES, pH 7.5, 0.2 mM CaCl_2_, 0.2 mM ATP, 0.5 mM DTT) or F-buffer (27.5 mM HEPES, pH 7.5, 0.2 mM CaCl_2_, 25 mM KCl, 1 mM EGTA, 4 mM MgCl_2_, 0.7 mM ATP, 0.5 mM DTT) in the presence of either ∼65 nM 19S regulatory particle or 50–100 nM 20S core particle in 125 µl total volume. The reactions were submitted to high-speed centrifugation (70,000 rpm for 30 min in a TL100 ultracentrifuge), followed by SDS-PAGE and SYPRO Ruby (Bio-Rad, Hercules CA) staining of the total reaction (T), supernatant (S) and pellet (P). Note that the supernatant fractions were taken from the top of the centrifugation reactions.
